# Mobility level and factors affecting mobility status in hospitalized patients admitted in single-occupancy patient rooms

**DOI:** 10.1186/s12912-023-01648-4

**Published:** 2024-01-02

**Authors:** Laura Schafthuizen, Monique van Dijk, Joost van Rosmalen, Erwin Ista

**Affiliations:** 1 Department of Internal Medicine, section Nursing Science, Erasmus University Medical Center, Rotterdam, The Netherlands; 2 Department of Biostatistics, Erasmus University Medical Center, Rotterdam, the Netherlands; 3 Department of Epidemiology, Erasmus University Medical Center, Rotterdam, the Netherlands

**Keywords:** Mobility, Hospitalization, Nursing, Functional decline

## Abstract

**Background:**

Although stimulating patients’ mobility is considered a component of fundamental nursing care, approximately 35% of hospitalized patients experience functional decline during or after hospital admission. The aim of this study is to assess mobility level and to identify factors affecting mobility status in hospitalized patients admitted in single-occupancy patient rooms (SPRs) on general wards.

**Methods:**

Mobility level was quantified with the Johns Hopkins Highest Level of Mobility Scale (JH-HLM) and EQ-5D-3L. GENEActiv accelerometer data over 24 h were collected in a subset of patients. Data were analyzed using generalized ordinal logistic regression analysis. The STROBE reporting checklist was applied.

**Results:**

Wearing pajamas during daytime, having pain, admission in an isolation room, and wearing three or more medical equipment were negatively associated with mobilization level. More than half of patients (58.9%) who were able to mobilize according to the EQ-5D-3L did not achieve the highest possible level of mobility according to the JH-HLM. The subset of patients that wore an accelerometer spent most of the day in sedentary behavior (median 88.1%, IQR 85.9–93.6). The median total daily step count was 1326 (range 22-5362).

**Conclusion:**

We found that the majority of participating hospitalized patients staying in single-occupancy patient rooms were able to mobilize. It appeared, however, that most of the patients who are physically capable of walking, do not reach the highest possible level of mobility according to the JH-HLM scale. Nurses should take their responsibility to ensure that patients achieve the highest possible level of mobility.

**Supplementary Information:**

The online version contains supplementary material available at 10.1186/s12912-023-01648-4.

## Background

Approximately 35% of hospitalized patients experience functional decline during or after hospital admission [1]. Abdulaziz et al. (2016) defined functional decline as “a reduction in ability to perform self-care activities of daily living (ADL) because of a decrement in physical or cognitive functioning” [2]. Functional decline may lead to reduced muscle weakness, pressure ulcers, fall risk, higher incidence of pneumonia, delirium, and venous thromboembolism [3–7]. Apart from these adverse consequences, functional decline could also lead to longer stay, institutionalization, and mortality [8, 9]. Several studies have shown that stimulating patients’ mobility during hospitalization reduces length of stay and has a positive effect on preventing functional decline [10, 11]. Although the literature documents the beneficial effects of early mobilization, in practice it appears that patients admitted to general wards spend the greater part of their hospital stay in bed [12]. Also, it has been estimated that only one third of older patients returned home at (or above) their premorbid level of function after an acute hospitalization [13].

Stimulating patients’ mobility is considered a component of fundamental nursing care, aimed at maintaining at least at the same levels of functioning as prior to hospital admission [11, 14]. However, earlier research reported several barriers from nurses’ perspectives to achieve this goal, such as insufficient skills to mobilize patients, the long time it takes to mobilize patients, and fall concern [15, 16].

Most studies about mobilization in hospitalized patients concerned patients staying in multi-bedded rooms [3, 7, 17, 18]. Single-occupancy patient rooms (SPRs) are a core feature in many new hospitals; and many benefits of SPRs on infection control, patient privacy, and control of light and sound have been described [19, 20]. Nevertheless, a disadvantage of SPRs may be that mobilization is not a natural part of the recovery process when patients have all they need in their rooms [21].

The aim of this study is to assess mobility level and to examine factors affecting mobility status in hospitalized patients admitted in SPRs on general wards.

## Methods

### Design

In a prospective cross-sectional study, we studied mobility level in hospitalized patients admitted in SPRs, and factors affecting mobility status. Furthermore, in a subset of patients we measured activity level during 24 h with the use of an accelerometer. The Medical Ethics Review Board of Erasmus MC approved the study protocol (MEC-2017-1103). Reporting of this study is according to the ‘Strengthening the Reporting of Observational Studies in Epidemiology’ (STROBE) Statement (see supplementary file 1) [22].

### Setting and participants

In May 2018, the Erasmus University Medical Center was relocated to a newly built hospital with exclusively SPRs and features such as an en-suite bathroom and the possibility for patients to control lighting and opening of windows themselves. Adult patients able to provide informed consent, accommodated in either internal medicine or surgical wards, were eligible for inclusion in this study. Excluded were patients from intensive care units and stroke units, as well as patients diagnosed with a delirium, confusion or reduced level of consciousness.

### Measures

#### The Johns Hopkins highest level of mobility (JH-HLM) scale

The Johns Hopkins Highest level of Mobility (JH-HLM) scale is an 8-point ordinal scale to categorize the highest-level of mobility a patient achieves. The mobility level is scored as follows: 1 = lying in bed, 2 = bed activities, 3 = sitting at edge of bed, 4 = transferring to chair/commode, 5 = static standing for at least 1 min, 6 = walking at least 10 steps, 7 = walking at least 7.5 m, and 8 = walking at least 75 m [23]. Previous research showed that the JH-HLM scale has excellent test–retest reliability (0.91) and inter-rater reliability (0.99) when applied by nurses [23]. In earlier research, the JH-HLM scale was used to document actual mobility levels, set mobility goals, and served as a tool to assess patient mobility and functional limitation in the hospital setting [23]. In our study, nursing students applied the JH-HLM scale to document actual mobility levels by asking patients what their highest level of mobility was on a scale from 1 to 8.

#### Accelerometry

Physical activity level in a subset of patients was measured with the tri-axial, wrist-worn GENEActiv accelerometer (Activinsights, Cambs, UK). It records acceleration in three planes with the acceleration due to gravity subtracted (unit milli-g) [24]. The GENEActiv accelerometer is fully waterproof and records data for up to one month without the need to charge. To conform with hygiene guidelines, the accelerometer was attached with disposable straps to a patient’s wrist. Total physical activity and step count was measured for a continuous twenty-four-hour period. The GENEActiv accelerometer proved to be valid, reliable and feasible in previous studies measuring physical activity in hospitalized patients [25–27]. We considered patients as ‘physically active’ if they had spent at least 20 min in moderate physical activity during the twenty-four-hour period [26].

#### EQ-5D-3 L

The Dutch versions of the EQ-5D-3L and the EQ-VAS served to assess the participants’ health state [28]. The EQ-5D-3L encompasses five dimensions of health state: ‘mobility’, ‘self-care’, ‘usual activities’, ‘pain/discomfort’, and ‘anxiety/depression’. The items are assessed with a three-level scale (no/some/extreme problems). Regarding the dimension ‘mobility’, the three response options are: I have no problems in walking about, I have some problems in walking about, I am confined to bed. The sum of all dimensions results in a health status index score ranging from −0.329 (worst possible health state) to 1 (perfect health) according to the Dutch EQ-5D tariff [29]. The EQ-VAS measures self-reported current health on a visual analogue scale from 0 (worst imaginable health state) to 100 (best imaginable health state). The EQ-5D-3L and EQ-VAS have shown to be valid, reliable and responsive in many situations and populations [28].

#### Patient demographic and clinical information

To assess factors affecting mobility status, we extracted additional information from electronic patient records including: age, sex, reason for admission, and length of total hospital stay. Information about the use of medical equipment such as drains, lines, and catheters was collected too, because these may hinder physical activity. We also recorded whether patients were admitted in isolation rooms. Previous research has demonstrated that wearing pajamas during daytime negatively affects mobility [30]. Therefore, we also recorded whether patients were wearing pajamas at the time the questionnaires were administered. In addition, we recorded whether patients used mobility aids such as a wheelchair or crutches.

### Data collection

Nursing students who had completed protocol training and reliability checks by the research coordinator (EI) collected data in two periods: from February 2020 to March 2020  and from March 2021 to June 2021. They visited the selected wards and first asked the nurse in charge which patients were eligible. These patients were invited to participate in the study, and if willing to participate provided informed consent. In this way, a convenience sample was obtained. The nursing students or, if possible, patients themselves, entered the answers of the JH-HLM scale, the EQ-5D-3L, as well as information about wearing pajamas during daytime and the use of mobility aids. Both questionnaires were collected once on a random day in the afternoon. Patients were asked to reflect on their mobility level at the moment the questionnaires were administered. We compared the mobility level measured with the JH-HLM scale to the mobility status assessed using the EQ-5D-3L to assess whether patients who are physically capable of walking actually mobilize to this extent. The EQ-5D-3L provides a more global reflection of the level of mobility with only three answer options (no/some/extreme), whereas the JH-HLM is more specific with eight categories. In addition, a randomly chosen subset of twenty-one patients were asked to wear a GENEActiv accelerometer on their non-dominant wrist for twenty-four hours. The GENEActiv accelerometer was placed by nursing students on the day the JH-HLM and EQ-5D-3L was administered, and was removed 24 h later. Patients were instructed not to remove the accelerometer intermediately during these 24 h. If medical procedures (e.g., MRI scan) required removal of the accelerometer, patients were instructed to remove the accelerometer just before start of the procedure and to put it back on immediately thereafter. All data were collected using an online form and were stored on a secured server and retrieved with Microsoft® Excel 2016.

### Statistical analysis

Categorical data are presented as numbers and percentages. Normally distributed continuous variables are presented as mean and standard deviation (SD); non-normally distributed variables as median and interquartile range (IQR). The data of the two periods were combined. We created three groups according to the JH-HLM scale assessment. Group 1: represents low mobility, i.e., category 1 to 5 of the JH-HLM scale; group 2: represents intermediate mobility, i.e., category 6 and 7 of the JH-HLM scale; group 3: represents high mobility, i.e., category 8. Factors added in the model were pain intensity according to EQ-5D-3L, room type (isolation room or not), number of medical devices (0 to 2 or more than 3), ward type (medical or surgical), and age. The significance of factors explaining level of mobility was assessed using generalized ordinal logistic regression. Ordinal logistic regression is a regression technique for ordered categorical outcomes such as the grouped JH-HLM scale. This technique makes the parallel lines (proportional odds) assumption, which implies that the odds ratio of an explanatory variable for the outcome being at least category *j* does not depend on the value of *j*. Under the proportional odds assumption, only a single coefficient is estimated for each category of an explanatory variable [31]. We used generalized ordinal logistic regression with a partial proportional odds model specification to be able to relax the parallel lines/proportional odds assumption for some of the explanatory variables. With the function ‘gologit2’ in STATA, the autofit procedure was used to determine for which variables the proportional odds assumption was rejected [31]. The level of statistical significance was set at *p* < 0.05.

The health status index scores EQ-5D-3L were calculated with R-package ‘EQ5D’ (https://cran.r-project.org/web/packages/eq5d/index.html). Activity characteristics, including step count, were derived from raw accelerometer data and processed using the open-source R-package ‘GENEAclassify’, version 1.5.2 (https://cran.r-project.org/web/packages/GENEAclassify/GENEAclassify.pdf). All other data were analyzed using IBM SPSS Statistics for Windows version 25.0 (IBM Corp., Armonk, NY) and STATA 15.0.

## Results

**Fig. 1:**
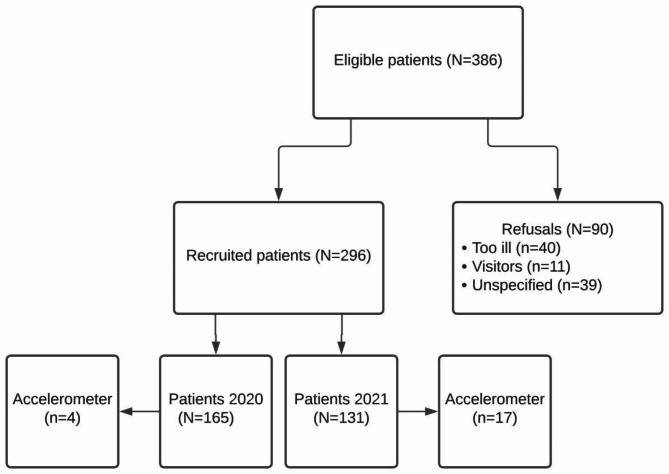
Flow chart

Three hundred and eighty-six patients met eligibility criteria and were invited to participate. Ninety of them (23.3%) declined participation for various reasons, such as having visitors, being too sick, reluctance to having a student entering the room (for fear of COVID-19) or declining to wear an accelerometer. Eventually, 296 (76.7%) patients participated; 165 in the period from February 2020 to March 2020, and 131 in the period from March 2021 to June 2021. Twenty-six of those wished to participate anonymously; therefore, additional demographic information of these patients could not be retrieved. Accelerometry data were collected in twenty-one patients. The flowchart in Fig. 1 details the inclusion flow.

### Demographic data

The overall median age was 60 (IQR 49–68) years. The proportion of surgical patients was 42.2%. Sixty-two patients (20.9%) stayed in an isolation room, and were not allowed to leave the room at the time the questionnaires were administered. Twenty (6.8%) patients were confined to bed according the EQ-5D-3 L. Almost half of the patients wore a pajama during daytime (47.6%). The median EQ-5D- L index score was 0.775 [0.520–0.861]. Table 1 lists all demographic and clinical characteristics.

**Table 1:** Demographic and clinical characteristics of included patients

Demographic and clinical characteristics	Total patients (n = 296)
Age in years *	60 [49–68]
Male, N (%)	164 (59.4)
Admission for, N (%)	
Surgery	111 (42.2)
Internal Medicine	152 (57.8)
Days between questionnaire and surgery*	4 [1–9]
Days between questionnaire and day of admission*	5 [2–11]
Isolation Room, N (%)	62 (20.9)
Length of stay in days*	10 [6–23]
Wearing pajamas, N (%)	141 (47.6)
Walking aid, N (%):	
- Canes	5 (1.7)
- Walker	36 (12.2)
- Crutches	3 (1.0)
- Supported by a person	11 (3.7)
- None	243 (82.1)
EuroQol-5D-3L index*	0.775 [0.520–0.861]
**Mobility**, N (%)	
I have no problems in walking about	153 (51.7)
I have some problems in walking about	123 (41.6)
I am confined to bed	20 (6.8)
**Self-care**, N (%)	
I have no problems with self-care	197 (66.6)
I have some problems washing or dressing myself	78 (26.4)
I am unable to wash or dress myself	21 (7.1)
**Usual activities**, N (%)	
I have no problems with performing my usual activities	145 (49.0)
I have some problems with performing my usual activities	106 (35.8)
I am unable to perform my usual activities	45 (15.2)
**Pain/discomfort**, N (%)	
I have no pain or discomfort	122 (41.2)
I have moderate pain or discomfort	166 (45.9)
I have extreme pain or discomfort	37 (12.5)
**Anxiety/depression**, N (%)	
I am not anxious or depressed	208 (70.3)
I am moderately anxious or depressed	77 (26.0)
I am extremely anxious or depressed	10 (3.4)
EuroQol-VAS*	60 [44–70]
**Medical equipment**, N (%)	
- One type of medical equipment	142 (54.0)
- Two types of medical equipment	40 (15.2)
- Three types of medical equipment	10 (3.8)
- Four types of medical equipment	3 (1.1)

*median [IQR]

#### Medical equipment

Of the twenty-six patients who participated anonymously it was obviously not known whether they had medical equipment. Sixty-eight of the remaining 263 (25.9%) did not have any medical equipment. One hundred and forty-two (48.0%) patients had one medical equipment, primarily a peripheral intravenous catheter. Forty (15.2%) patients had two types of medical equipment. The remaining thirteen patients (4.9%) had more than three types of medical equipment (Table 1).

### JH-HLM vs. EQ-5D-3L

In Table 2, mobility level measured with the JH-HLM scale is set against mobility status according the EQ-5D-3L. Out-of-bed mobility (JH-HLM scale category 6, 7 and 8) was reported in 260 (89.3%) patients. Thirty patients (10.7%) could be classified in group 1 (category 1 to 5), 150 (51.5%) in group 2 (category 6 and 7), and 110 (37.8%) in group 3 (category 8) patients. The majority of patients (58.9%) who according to the EQ-5D-3L have no problems in walking about did not reach the highest possible category on the JH-HLM scale. Supplementary Table 2 presents the characteristics for patients divided into JH-HLM groups 1, 2, and 3. The main differences between the active and passive groups were the median length of stay (in days), with seventeen and sixteen days for groups 1 and 2, respectively, and eight days in group 3. Median current health status according the EQ-VAS is higher in group 1 than those in groups 2 and 3, with a median score of 85 compared to 60 and 65. All patients in group 1 were wearing pajamas during the day, while in group 2, nearly half of the patients (49.4%) did so, and in group 3, less than a third of patients (30.6%).

**Table 2:** Association between JH-HLM and Eq. 5D

	I have no problems in walking about	I have some problems in walking about	I am confined to bed
JH-HLM category 1 to 5, n (%)	4 (2.6)	11 (9.2)	16 (80)
JH-HLM category 6 and 7, n (%)	85 (56.3)	61 (50.8)	4 (20)
JH-HLM category 8, n (%)	62 (41.1)	48 (40)	0 (0)

### Factors affecting level of mobility

To identify which of the studied factors were associated with mobility level, we conducted a generalized ordinal logistic regression analysis (Table 3). The proportional odds assumption was rejected for variables ‘isolation room’ and ‘number of medical equipment’, and two odds ratios are reported for each of these two variables, to represent respectively the thresholds between group 1 and group 2 and those between group 2 and group 3. For comparisons between groups, wearing pajamas (OR = 1.89, 95% CI 1.10 to 3.23, *p* = 0.021) and having pain (no pain or discomfort versus extreme pain or discomfort: OR = 0.30, 95% CI 0.12 to 0.71, *p* = 0.006, moderate pain or discomfort versus extreme pain or discomfort: OR = 0.27, 95% CI 0.11 to 0.62, *p* = 0.002) were more likely in patients with a lower JH-HLM scale score. Staying in an isolation room was associated with having a low or intermediate level of mobility (group 1 or 2) versus a high level of mobility (OR 11.15, 95% CI 4.25 to 29.24, *p* < 0.001), but not with the distinction between a low mobility (group 1) and an intermediate or high mobility (group 2 or 3). Patients with fewer than three types of medical equipment were less likely to have a low level of mobility (group 1) (OR = 0.13, 95% CI 0.04 to 0.44, *p* = 0.001).

**Table 3:** Generalized ordinal logistic regression with JH-HLM score as outcome

Parameter	Odds ratio	95% CI	*p*-value
*Group type*			
[Group 1 or 2 (low or intermediate mobility) vs. group 3 (high mobility)]			
*Wearing pajamas during daytime*			
Wearing pajamas	1.89	1.10 to 3.23	0.021
Not wearing pajamas	reference ^a^		
*Pain*			
I have no pain or discomfort	0.30	0.12 to 0.71	0.006
I have moderate pain or discomfort	0.27	0.11 to 0.62	0.002
I have extreme pain or discomfort	reference ^a^		
*Room type* Admitted in isolation room Admitted in standard room	11.15 reference ^a^	4.25 to 29.24	< 0.001
*Number of medical equipment* < 3 medical equipment ≥ 3 medical equipment	0.69 reference ^a^	0.20 to 2.39	0.554
*Ward type*			
Surgery	1.67	0.94 to 2.97	0.080
Internal medicine	reference ^a^		
*Age*	1.00	0.98 to 1.01	0.729
*Group type*			
[Group 1 (low mobility) vs. group 2 or 3 (intermediate or high mobility)]			
*Wearing pajamas during daytime*			
Wearing pajamas	1.89	1.10 to 3.23	0.021
Not wearing pajamas	reference ^a^		
*Pain*			
I have no pain or discomfort	0.30	0.12 to 0.71	0.006
I have moderate pain or discomfort	0.27	0.11 to 0.62	0.002
I have extreme pain or discomfort	reference ^a^		
*Room type* Admitted in isolation room Admitted in standard room	0.60 reference ^a^	0.16 to 2.26	0.446
*Number of medical equipment* < 3 medical equipment ≥ 3 medical equipment	0.13 reference ^a^	0.04 to 0.44	0.001
*Ward type*			
Surgery	1.67	0.94 to 2.97	0.080
Internal medicine	reference ^a^		
*Age*	1.00	0.98 to 1.01	0.729

^a^ Reference category

Group 1 = JH-HLM scale, category 1 to 5

Group 2 = JH-HLM scale, category 6 and 7

Group 3 = JH-HLM scale, category 8

### GENEActiv accelerometer

We collected accelerometer data in a randomly selected subset of twenty-one patients. Their median age was 62 (IQR 54–66), 15 (71.4%) were male, and 5 (23.8%) were surgical patients. Nineteen (90.5%) of them reported they could walk without assistance or walking aid. One patient (4.8%) was confined to bed and one patient (4.8%) mobilized with a walker.

The median activity time was 2.2 h/day (IQR 1.25–2.7). The greater part of the day (median 88.1%, IQR 85.9–93.6) was spent in sedentary behavior. The median total daily step count was 1326 (min 22, max 5362). Five (23.8%) patients were considered ‘physically active’, defined as spending at least 20 min/day in moderate physical activity. None of the patients had been vigorously active during the day. In supplemental Table 3 we divided patients with an accelerometer into two groups (< 1500 steps and ≥ 1500 steps). More patients in the first group scored category 7 on the JH-HLM; i.e., eight (61.5%), while five patients in the other group (62.5%) scored category 8. More patients in the first group were wearing pajamas during the day; i.e., nine (69.2%) compared with two (25%) in the other group.

## Discussion

In this study, we found that the majority of hospitalized patients staying in single-occupancy patient rooms were able to mobilize. It appeared, however, that most of the patients who are physically capable of walking, do not reach the highest possible level of mobility according to the JH-HLM scale. A possible explanation could be that patients admitted in SPRs with en-suite bathrooms, a tablet and television, and extended visiting hours, do not feel the urge to go outside their room. The subset of patients equipped with an accelerometer for 24 h spent most of this time in sedentary behavior. Generalized ordinal logistic regression analysis revealed that wearing a pajama in daytime, having (extreme) pain or discomfort, staying in an isolation room, and having three or more types of medical equipment are factors that are negatively associated with mobilization level. As suggested in earlier research, mobilization in SPRs may be hampered because the patients have all they need in their rooms [21]. In the observational study by Kuys et al., there were no significant differences in mean time spent lying in bed (in minutes) between patients in single-occupancy rooms and multi-bedded rooms, with respective values of 294 (SD 121) and 302 (SD 96) minutes [32]. Other studies have reported similar findings, with patients spending most of their time in bed during the day, ranging from 57.4 to 90.4% [7, 26, 32].

Similar to the findings of So et al., we found that having lines and catheters inserted are important barriers for optimizing physical activity [33]. Therefore, considering the possibility of early removal of catheters and intravenous lines deserves attention. Also, in line with our findings, wearing pajamas in the daytime, so-called ‘pajama paralysis’ according to Brian Dolan, seems to negatively affect mobilization [30]. Experiencing pain can also be a barrier to being physically active [34]. On the other hand, in a previous study early mobilization after surgery resulted in less postoperative pain [35]. Fear of experiencing pain during mobilization and ineffective pain medication are described as limiting factors from a patient’s perspective [36]. Nurses can help patients overcome fear of pain by providing clear information about how pain medication, as well as other non-pharmacological modalities such as breathing and relaxation techniques, can be effective in relieving pain during mobilization [37–39].

In general, nurses are key in supporting mobilization as they can observe and guide patients [40]. However, in practice it appears that most of the patients admitted to medium care wards spend the greater part of the day in their room, which also may contribute to functional decline [41]. Resnick et al. applied numerous approaches to increase physical activity but no single approach was found effective [42]. However, the systematic review by Foubert al. identified four studies that reported effective interventions to increase mobility (level, distance, or frequency) [43]. First, Klein et al. reported a higher patient mobility level on the JH-HLM scale after implementation of an individualized, nurse-directed, patient mobility program involving staff education, documentation, audit, and feedback [3]. Second, Hoyer et al. developed and tested a multidisciplinary mobility promotion quality improvement project, aimed at mobilizing general medicine patients three times a day and to set daily goals to increase mobility [44]. This resulted in improved mobility and a shorter length of stay. Third, an increasing mobilization was achieved in medical-surgical patients who participated in a nurse-led mobility program consisting of education for nurses, providing a gait belt in every patient room, and hiring a mobility coordinator [45]. Fourth, King et al. found a significant increase in mobilization frequency and ambulation distance after an intervention in a pilot study in a general medical unit [46]. The intervention comprised five components: skills training for nurses in helping patients to ambulate, communication tools, ambulation resources (equipment), ambulation pathways, and ambulation culture to establish nurse ownership.

Kok et al. described the implementation of function focused care (FFC) in a Dutch hospital [47]. FFC is a philosophy of care in which nurses work with patients to optimize function and physical activity during all care interactions [42]. FFC has proven to be feasible for the Dutch hospital setting. As the authors put it, team involvement, nursing leadership during implementation, and the involvement of patients and their family is needed to optimize future implementation of FFC in Dutch hospitals.

We used actigraphy (GENEActiv) to assess activity in a more objective way. The importance of activity was emphasized in earlier research, which found an association between step count and shortened length of stay [48, 49]. We found a median total daily step count of 1326, most of which was performed in short time frames and at a low intensity. These findings are comparable to the results of Rice et al., who reported a step count of 926 steps per day [50]. Fisher et al. found that patients admitted to an Acute care for Elders unit who increased their daily step count by 600 steps daily were discharged on average 1.7 days earlier than patients who did not [51].

### Strength and limitations

A strength of this study is that in a large sample of patients diverse factors potentially affecting mobilization were identified. Still, several limitations of the study need to be considered. First, almost 40% of the patients scored the highest possible mobility score on the JH-HLM scale, which indicates a ceiling effect. The AMEXO scale appeared to be more sensitive to detect changes in mobility in hospitalized surgical patients [52]. Second, we do not know how many patients refused to get out of bed and mobilize with the nurse or physiotherapist. Third, during the first measurement period, the COVID-19 pandemic started. While the included wards were not designated COVID-19 wards, it may be assumed that the level of activity of included patients was affected by the pandemic because in some wards they were forbidden to leave the room. Fourth, response bias may have occurred since we used questionnaires in which patients self-scored their mobility level [53]. Finally, it is a limitation that this is an cross-sectional study without a control group. Our results would be more reliable if we had performed this study in a setting with multi-bedded rooms.

## Conclusion

This study provided insight into potentially modifiable factors affecting mobility in hospitalized patients staying in single-patient rooms, and identified several targets for nursing care to enhance mobility. These targets include discouraging patients to wear a pajama in the daytime, and early removal of medical devices such as catheters and intravenous lines. Further research is needed to explore other factors potentially associated with low mobility, such as poor collaboration between physiotherapists and nurses, barriers from patients’ perspectives, and to implement nursing interventions such as Function Focused Care.

### Electronic supplementary material

Below is the link to the electronic supplementary material.

Supplementary Material 1

Supplementary Material 2

Supplementary Material 3

## Data Availability

The datasets used and analyzed during the current study are available from the corresponding author on reasonable request.
