# Supplementary material for: Mobility level and factors affecting mobility status in hospitalized patients admitted in single-occupancy patient rooms
Source: BMC Nurs. 2024 Jan 2;23:11. doi: 10.1186/s12912-023-01648-4 (PMC10759502; doi:10.1186/s12912-023-01648-4)
Supplement: Supplementary file 2 — Supplementary Material 2 [file 12912_2023_1648_MOESM2_ESM.docx]

**Supplemental table 3**

|  | **Less than 1500 steps (N=13)** | **Equal to or more than 1500 steps (N=8)** |
| --- | --- | --- |
| Male, N(%) | 9 (69.2) | 6 (75) |
| Surgical patients, N(%) | 3 (23.1) | 2 (25) |
| EQ-5D-3L Mobility |  |  |
| *I have no problems in walking about* | 9 (69.2) | 7 (87.5) |
| *I have some problems in walking about* | 3 (23.1) | 1 (12.5) |
| *I am confined to bed* | 1 (7.7) | 0 |
| JH-HLM |  |  |
| *Lying in bed* | 0 | 0 |
| *Bed activities* | 0 | 0 |
| *Sit at edge of bed* | 1 (7.7) | 0 |
| *Move to chair* | 0 | 0 |
| *Standing* | 0 | 0 |
| *Walking at least 10 steps,* | 1 (7.7) | 2 |
| *Walking at least 7.5 meters* | 8 (61.5) | 1 (12.5) |
| *Walking at least 75 meters* | 3 (23.1) | 5 (62.5) |
| Wearing pajamas during daytime, N(%) | 9 (69.2) | 2 (25) |
| Number of medical equipment |  |  |
| *One type of medical equipment* | 12 (92.3) | 3 (37.5) |
| *Two types of medical equipment* | 1 (7.7) | 1 (12.5) |
| *Three types of medical equipment* | 0 | 1 (12.5) |
| *Four types of medical equipment* | 0 | 0 |
| EuroQol-5D-3L index* | 0.843 [0.725-1.000] | 0.843 [0.530-0.922] |

*median [IQR]
