# Supplementary material for: Mobility level and factors affecting mobility status in hospitalized patients admitted in single-occupancy patient rooms
Source: BMC Nurs. 2024 Jan 2;23:11. doi: 10.1186/s12912-023-01648-4 (PMC10759502; doi:10.1186/s12912-023-01648-4)
Supplement: Supplementary file 3 — Supplementary Material 3 [file 12912_2023_1648_MOESM3_ESM.docx]

Supplemental table 2. Patient characteristics divided by JH-HLM categories

| **Demographic and clinical characteristics** | **JH-HLM 1-5**  **(N=4)** | **JH-HLM 6-7**  **(N=85)** | **JH-HLM 8**  **(N=62)** |
| --- | --- | --- | --- |
| Age in years * | 52 [45-61] | 60 [48-65] | 57 [47-65] |
| Male, N (%) | 1 (25) | 50 (58.8) | 41 (66.1) |
| Admission for, N (%) |  |  |  |
| Surgery | 3 (75) | 21 (24.7) | 22 (35.5) |
| Internal Medicine | 1 (25) | 53 (62.3) | 36 (58.1) |
| Days between questionnaire and surgery* | 1 [1-1] | 4 [1-11] | 6 [2-9] |
| Days between questionnaire and day of admission* | 3 [2-9] | 6 [2-14] | 4 [2-7] |
| Isolation room, N (%) | 1 | 37 | 6 |
| Length of stay in days* | 17 [8-36] | 16 [6-29] | 8 [5-16] |
| Wearing pajamas, N (%) | 4 (100) | 42 (49.4) | 19 (30.6) |
| Walking aid, N (%): |  |  |  |
| - Canes | 0 | 0 | 0 |
| - Walker | 0 | 0 | 2 (3.2) |
| - Crutches | 0 | 0 | 0 |
| - supported by a person | 0 | 1 (1.2) | 1 (1.6) |
| - None | 4 (100) | 82 (96.5) | 59 (95.2) |
|  |  |  |  |
| **EuroQol-5D-3L index*** | 0.949 [0.808-1.000] | 0.843 [0.805-1.000] | 0.843 [0.723-1.000] |
| **Self-care,** N (%) |  |  |  |
| I have no problems with self-care | 4 (100) | 76 (89.4) | 53 (85.5) |
| I have some problems washing or dressing myself | 0 | 9 (10.6) | 8 (12.9) |
| I am unable to wash or dress myself | 0 | 0 | 1 (1.6) |
| **Usual activities,** N (%) |  |  |  |
| I have no problems with performing my usual activities | 3 (75) | 69 (81.2) | 40 (64.5) |
| I have some problems with performing my usual activities | 1 (25) | 14 (16.5) | 18 (29.0) |
| I am unable to perform my usual activities | 0 | 2 (2.4) | 4 (6.5) |
| **Pain/discomfort,** N (%) |  |  |  |
| I have no pain or discomfort | 3 (75) | 47 (55.3) | 31 (50) |
| I have moderate pain or discomfort | 1 (25) | 33 (38.8) | 25 (40.3) |
| I have extreme pain or discomfort | 0 | 5 (5.9) | 5 (8.1) |
| **Anxiety/depression,** N (%) |  |  |  |
| I am not anxious or depressed | 3 (75) | 67 (78.8) | 44 (71) |
| I am moderately anxious or depressed | 1 (25) | 17 (20) | 16 (25.8) |
| I am extremely anxious or depressed | 0 | 1 (1.2) | 2 (3.2) |
| EuroQol-VAS* | 80 [60-90] | 60 [40-70] | 65 [50-75] |
| **Medical equipment,** N (%) |  |  |  |
| - One type of medical equipment | 1 (25) | 51 (60) | 37 (59.7) |
| - Two types of medical equipment | 2 (50) | 8 (9.4) | 3 (4.8) |
| - Three types of medical equipment | 1 (25) | 1 (1.2) | 2 (3.2) |
| - Four types of medical equipment | 0 | 1 (1.2) | 0 |

*median [IQR]
